# Resveratrol and temozolomide induce apoptosis and suppress proliferation in glioblastoma cells via the apoptotic signaling pathway

**DOI:** 10.1590/acb405525

**Published:** 2025-08-08

**Authors:** Görkem Tutal Gürsoy, Mehmet Cudi Tuncer, İlhan Özdemir

**Affiliations:** 1 Ankara City Hospital - Department of Neurology - Ankara - Turkey.; 2 Dicle University - Faculty of Medicine - Department of Anatomy - Diyarbakir - Turkey.; 3 Atatürk University - Faculty of Medicine - Department of Gynecology and Obstetrics - Erzurum - Turkey.

**Keywords:** Resveratrol, Temozolomide, Apoptosis, Glioblastoma, Cell Survival

## Abstract

**Purpose:**

Glioblastoma (GBM) is the most common primary brain tumor in the central nervous system. Studies revealing the molecular mechanisms regulating GBM pathogenesis are currently limited. This study aimed to investigate the expression of genes responsible for the apoptotic pathway (p21, p27, p53) after separate and combined application of the natural components resveratrol (Res) and temozolomide (TMZ) in the GBM cell line (U118).

**Methods:**

In this study, the GBM cell line U118 was used. Apoptotic activation of Res and TMZ via the p21, p27, p53 signaling pathway was evaluated by quantitative reverse transcription polymerase chain reaction and TaLi cytometry. Cell viability was also assessed using the MTT assay.

**Results:**

Res and TMZ inhibited the proliferation and migration of U118 cells. Additionally, Res induced apoptosis by arresting the cell cycle. Moreover, Res treatment upregulated the expression of p27 and p53, which are associated with apoptosis, while it significantly downregulated the expression of the p21 gene.

**Conclusion:**

These results indicated that Res and TMZ suppressed the proliferation of GBM cells through apoptotic pathways. Together, Res and TMZ may represent a promising combination for suppressing tumors through apoptotic mechanisms.

## Introduction

Glioblastoma (GBM) is the most common primary brain tumor in the central nervous system, accounting for approximately 45-50% of all gliomas. Despite modern diagnostic and therapeutic approaches such as surgical resection, radiation, and chemotherapy, patients have an average survival of 12 to 15 months. However, after surgical removal of tumor tissue, tumor recurrence often occurs within 1 cm of the resection cavity. This is mostly related to migration of cells from tumor tissue to normal brain tissue during surgery^1^.

GBMs have been classified into four stages by the World Health Organization (WHO), based on histological, prognostic, and survival characteristics. Stage IV glioma, the most malignant class of GBM, is characterized by an extremely poor prognosis, with an average survival rate of only 3.3% at two years and 1.2% at three years. On the other hand, it has been reported that some low-grade gliomas may also progress to GBM. These are called secondary glioblastomas, while de novo GBM tumors are called primary glioblastomas^2^.

GBM formation is a complex, multistep process that includes cellular neoplastic transformation, resistance to apoptosis, loss of control of the cell cycle, angiogenesis, and acquisition of invasive properties. Nuclear factor-kappa B (NF-κB), reactive oxygen and nitrogen species, and specific microRNAs are key mediators of cancer progression. These mediators create a pro-tumorigenic response through changes in cell proliferation, cell lifespan, cellular senescence, DNA mutation rates, DNA methylation, and angiogenesis^3^.

Alkylating agents are the most used chemotherapeutic drugs in the treatment of GBM. GBM is classified as a WHO grade IV astrocytic tumor and represents the most aggressive form of primary brain malignancy. According to the 2021 WHO classification of central nervous system tumors, GBM is typically isocitrate dehydrogenase (IDH)-wildtype and exhibits features such as necrosis and/or microvascular proliferation. While some lower-grade gliomas (WHO grades II and III) may undergo malignant transformation into secondary glioblastomas, primary glioblastomas arise de novo and are classified as grade IV from the outset.

This updated classification emphasizes molecular profiling, such as IDH mutation status, to better define tumor subtypes and prognostic expectations^4^. It causes damage by adding a methyl group to the O6 position of guanine in the most critical region of DNA. This damage causes cancer cells to stop in the G2/M cell cycle and leads to apoptosis^5^. Temozolomide (TMZ) is frequently used in the treatment of recurrent glioma and has an antitumor effect. Treatment with TMZ causes O-6-methylguanine-DNA methyltransferase upregulation and increases cancer resistance. Therefore, while TMZ is used as a chemotherapeutic drug, it also paves the way for tumor recurrence^6^,^7^. Malignant gliomas are usually treated with TMZ, but tumor cells are resistant to this chemotherapy.

Nowadays, interest in resveratrol (Res) has increased greatly. It is known that Res has antioxidant and anti-inflammatory effects that play a role in the basis of many diseases. Its potential importance in the prophylaxis/treatment of chronic diseases such as cancer, heart diseases, diabetes, obesity, and aging-related eye diseases has been proven *in vitro* and *in vivo* 8. It is known that Res inhibits cellular events related to the three main steps of carcinogenesis-initiation, proliferation and metastasis-by inducing cell cycle arrest and apoptosis-mediated cell death. Its growth inhibitory effect on various cancer cells has been demonstrated in vitro. These include colon, prostate, breast, lymphoma and leukemia, cancer cells^9^,^10^. It has been reported that Res suppresses antitumor by strengthening the immune system^11^. In a cancer study, it has been reported that the Res analog HS-1793 enhances the function of radiotherapy by strengthening immunity in breast cancer^12^. However, the combined effect of Res with chemotherapy agents is very limited.

In this study, the anticancer activity of Res was investigated with TMZ, which is frequently used in the treatment of GBM.

## Methods

### Experimental workflow

**Figure 1:**
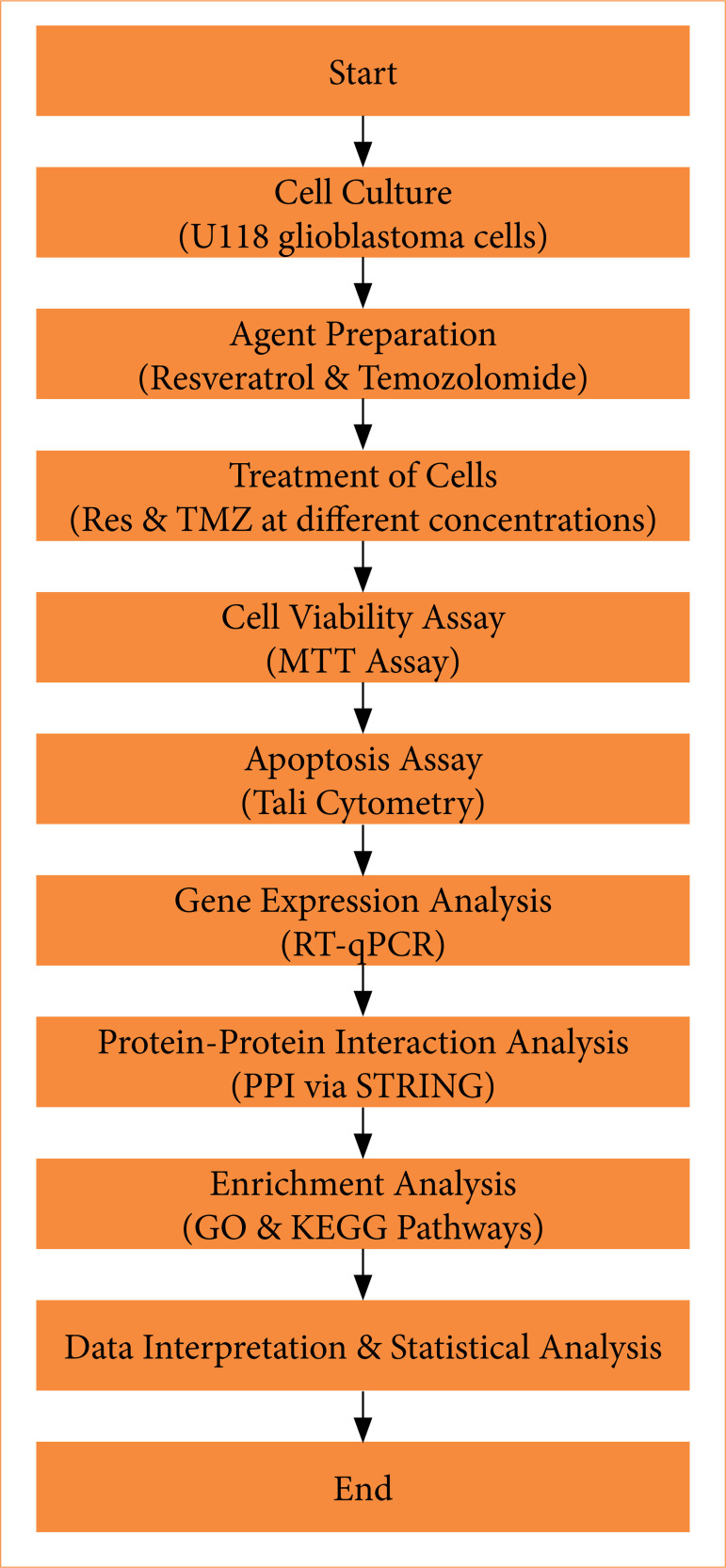
Res and TMZ experimental workflow in glioblastoma research. Res: resveratrol; TMZ: temozolomide; RT-qPCR: quantitative reverse transcription polymerase chain reaction.

Source: Elaborated by the authors.

The study followed a systematic experimental workflow. U118 GBM cells were cultured under controlled conditions and treated with Res and TMZ at varying concentrations. Cell viability was assessed using the MTT assay to determine cytotoxic effects. Apoptotic activity was evaluated through Tali cytometry, while gene expression changes in apoptotic markers (p21, p27, p53) were analyzed using quantitative reverse transcription polymerase chain reaction (RT-qPCR). Protein-protein interactions were examined via the STRING database, followed by functional enrichment analysis using GO and KEGG pathway tools. Finally, statistical analyses were conducted to validate the findings and interpret the biological significance of the results (Fig. 1).

### Agents

Res was dissolved in dimethyl sulfoxide (DMSO; SigmaChem Co., St. Louis, United States of America) to prepare a stock solution at concentration of 100 mM, and TMZ (3,4-dihydro-3-methyl-4-oxoimidazo [5,1-d]-as-tetrazine-8-carboxamide) (SigmaChem Co., St. Louis, United States of America) was dissolved in DMSO to prepare a stock solution at concentration of 1 mM for protection against light and stored at -20°C. MTT (3-[4,5-dimethylthiazol-2-yl]-2,5-diphenyl-tetrazolium bromide) was purchased from Sigma (SigmaChem Co., St. Louis, United States of America).

### Cell culture

The human U118 GBM cell line was used in the study. It was cultured in Dulbecco’s modified Eagle’s medium (DMEM) containing 10% fetal bovine serum (FBS), 3.5 mg/mL glucose, and 1% penicillin/streptomycin at 37°C temperature, 95% humidity and 5% CO_2_. The culture medium was changed every 24 hours until the cells reached the required majority. For the experiments, cells were treated with different concentrations of TMZ (5, 10, 25, 50, 75, 100, 250 and 500 μM) and Res (5, 10, 25, 50, 75, 100, 250, 500) for 24 and 48 h.

### Cell viability

MTT test was performed for cell survival (viability) analysis after incubation. For this purpose, the “Yellow tetrazolium MTT” test solution, prepared at the dose of 5 mg/mL, was pipetted into all wells at 20 µL/well. Then, the plates were left to incubate for 4 h. After the incubation, the medium in the wells was completely removed, and 200 µL ultra-puree DMSO (Merk, United States of America) was added to each well and waited in the incubator under dark conditions for 2-4 h. At the end of this period, the plates were read spectrophotometrically at 490 nm wavelengths with a Multiskan GO microplate reader (ThermoScientific, United States of America). The value obtained from the vehicle-treated control group was determined as a comparative viability rate based on 100% viability.

### Apoptotic assay

Apoptosis determination studies with Tali cytometer were performed according to the kit procedure using Tali Apoptosis Kit-Annexin V AlexaFluor 488 and PropidiumIodide (Life technologies). For the apoptosis determination study with the Tali cytometer, cells were planted in a 24-well plate with 1 mL of medium at approximately 5 × 104 cells/well. Depending on the proliferation status of the cells, they were incubated in a carbon dioxide incubator for one or two days. Application was made with the application doses determined in the previous study. At the end of the 48-h incubation period, the medium in the 24-well plates was removed. One-mL trypsin ethylenediamine tetraacetic acid (EDTA) was added to these wells, incubated for 10 minutes, and the cells were collected with an automatic pipette and placed in Eppendorf tubes. They were centrifuged at 700 rpm for 2 minutes. At the end of the centrifugation process, the upper trypsin and supernatant were completely removed, and the cells that settled at the bottom of the tube were used for analysis. After incubation, 25 µL of the mixture in the tubes was poured onto special slides prepared for Tali and read with the Tali apoptosis analysis program.

### Combination index calculation and synergism evaluation

To determine the nature of the interaction between Res and TMZ, combination index (CI) analysis was performed based on the Chou-Talalay method. U118 GBM cells were treated with Res, TMZ, and a fixed-ratio combination of both agents across a range of concentrations for 48 h. Cell viability was determined via MTT assay, and percentage inhibition was calculated. Interpolated dose-response curves for single agents were used to estimate the equivalent doses (Dx) needed to achieve the same effect as the combination doses. CI values were calculated using Eq. 1:


$$CI\;=\;(D1/Dx_{1})\;+\;(D2/Dx_{2})$$ (1)

where: D_1_ is the dose of Res used in combination treatment to achieve a specific percentage of cell inhibition in GBM U118 cells; D_2_ is the dose of TMZ used in combination treatment to achieve the same level of inhibition as D1; Dx_1_ is the dose of Res required to produce the same percentage of inhibition when used alone; Dx_2_ is the dose of TMZ required to produce the same percentage of inhibition when used alone.

D_1_ and D_2_ are the doses in combination, and Dx_1_ and Dx_2_ are the doses of each agent alone. CI < 1 indicates synergy, CI = 1 indicates an additive effect, and CI > 1 indicates antagonism. The results were visualized as a dose-CI curve and tabulated with interpretative categories.

### Quantitative reverse transcription polymerase chain reaction analysis

During the isolation phase, Purelink RNA mini kit (Thermo, United States of America) was used, and the kit protocol was followed. RNA samples (High-capacity cDNA reverse transcription kit, Applied Biosystem) were converted into cDNA according to the kit procedure. cDNA amounts were determined with Take3 Plate (Epoch Spectrophotometer System, Biotek). P21, p27 and p53 expression analysis were performed. Quantitative PCR analysis was performed with TaqMan probe mixed solution (Taqman Probe-based technology, Applied Biosystem). The Βeta actin gene was used as an endogenous control. Optical PCR plates were used for RT-qPCR analysis, and each group was studied in three replicates. Results were calculated with the 2-ΔΔCt method15 and are presented as fold change compared to the control group. In the study, the expression levels of P21, p27 and p53 pathway genes in control and treatment groups of GBM cells were analyzed by RT-qPCR method. The primers used to investigate the changes in the expression of these genes are given in Table 1, in 5’-3’ order.

**Table 1:** Primer sequences used for quantitative reverse transcription polymerase chain reaction analysis of target genes.

P21^Chip1^: F: GGCGTTTGGAGTGGTAGAAA, R: GACTCTCAGGGTCGAAAACG
P27^Kip1^: F: CCGGCTAACTCTGAGGACAC, R: TGGATCCAAGGCTCTAGGTG
P53: F: CACGAGCGCTGCTCAGATAGC, R: ACAGGCACAAACACGCACAAA
Βeta-Actin: F: CCTCTGAACCCTAAGGCCAAC, R: TGCCACAGGATTCCATACCC
GAPDH;F:CGGAGTCAACGGATTTGGTCGTAT, R:GCCTTCTCCATGGTGGTGAAGAC

### Protein-protein interaction

Protein-protein interaction (PPI) data were retrieved from the STRING database. The STRING database provides descriptions of PPIs, as well as confidence intervals for data scores. A confidence score greater than or equal to 0.4 was chosen to construct the interaction network of proteins with target genes.

### Enrichment analysis

Data on the functional annotation of genes and the canonical pathways associated with the strong connections established with these proteins were obtained using the ShinyGO 0.80 program.

### Gene ontologies enrichment analysis

Three types of gene ontologies (GO) were performed on possible target genes: cellular component (CC), biological process (BP), and molecular function (MF). The SRplot bio-informatics program was used to evaluate these data.

## Results

### Cytotoxic effect

The effects of Res on the proliferation of U118 GBM cells were determined by the MTT assay cell viability detection method. It was determined that control group cells increased for 48 h. It was observed that Res started to significantly reduce U118 cell numbers from the moment it was first applied. In the first step of the study, to determine the half-maximal inhibitory concentration (IC_50_) doses of Res and TMZ applications on GBM cell lines, U118 cells were treated with eight different agent doses in 5-500-µM concentration ranges for 48 h, and then the death rates were determined compared to the vehicle-treated control group using the MTT test. As a result of TMZ and Res application, it was observed that there was a significant decrease in cell viability depending on the dose increase.

### Apoptotic findings

**Figure 2:**
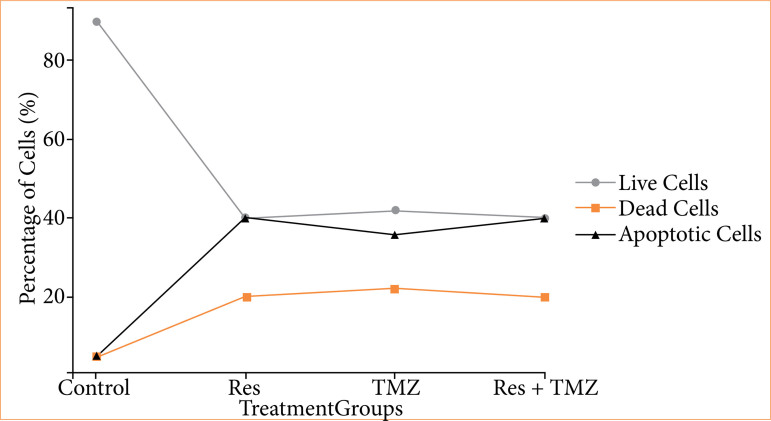
The effects of Res and TMZ on GBM cell viability and apoptosis. The x-axis represents the different treatment groups: Control, Res, TMZ, and Res + TMZ, while the y-axis indicates the percentage of cells. The green line represents live cells, showing a sharp decline from approximately 90% in the control group to around 40% in all treatment groups. This suggests that both Res and TMZ significantly reduced GBM cell viability. The red dashed line represents dead cells, increasing from around 5% in the control group to about 20% in the treatment groups. This increase indicates that both Res and TMZ contributed to cell death, though apoptosis played a more dominant role. The black dash-dotted line represents apoptotic cells, showing a significant rise from around 5% in the control group to approximately 40% in the Res and Res + TMZ groups. This highlights that Res strongly induced apoptosis, both alone and in combination with TMZ. Res: resveratrol; TMZ: temozolomide; GBM: glioblastoma.

Source: Elaborated by the authors.

**Figure 3:**
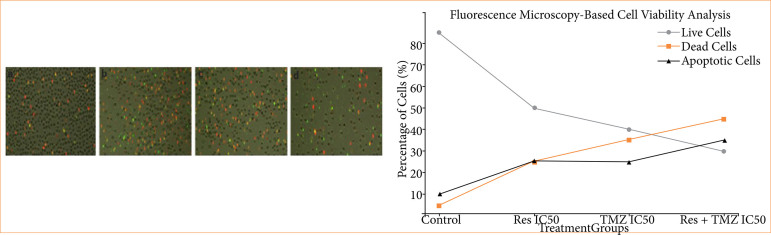
Combination of fluorescence microscopy images and corresponding cell viability data for U118 GBM cells treated with Res and TMZ at IC50 concentrations over a 48-h period. The top panel displays the fluorescence microscopy images under different treatment conditions: in the control group, a high density of live cells is seen with very few apoptotic and dead cells. With Res treatment at IC50, there is an increase in apoptotic cells, as indicated by the red staining, suggesting cell death induction. TMZ treatment at IC50 results in a further increase in apoptotic and dead cells, demonstrating a stronger cytotoxic effect. The combination of Res and TMZ at IC50 shows the highest proportion of apoptotic and dead cells, indicating a synergistic effect of the two treatments in inducing cell death. The bottom panel presents the quantitative analysis of cell viability based on fluorescence microscopy data. The percentage of live cells, represented by the green line, shows a significant decline from control to the combination treatment. The red dashed line represents the percentage of dead cells, which increases progressively with treatment, especially in the Res+TMZ group. The black dash-dotted line shows the increase in apoptotic cells, further emphasizing the apoptosis-inducing effect of the treatments. Res: resveratrol; TMZ: temozolomide; GBM: glioblastoma; IC50: half-maximal inhibitory concentration.

Source: Elaborated by the authors.

In the apoptotic staining performed with the Annexin V staining kit, it was observed that the cells undergoing apoptosis increased in Res + TMZ combination (Fig. 2). It was determined that the number of viable cells showed a statistically significant decrease compared to the control group. The effectiveness of Res in accelerating the apoptotic process was revealed (Fig. 3).

The overall trend suggests that Res and TMZ effectively inhibited GBM cell proliferation, primarily by inducing apoptosis rather than direct necrotic cell death. The combination of Res and TMZ did not appear to enhance cell death beyond individual treatments, implying that Res might have already maximized apoptotic activation when used alone.

These results highlight that both Res and TMZ significantly reduced GBM cell viability, with the combination treatment exhibiting the strongest cytotoxic and apoptotic effects.

### Combination index analysis of resveratrol and temozolomide co-treatment

**Figure 4:**
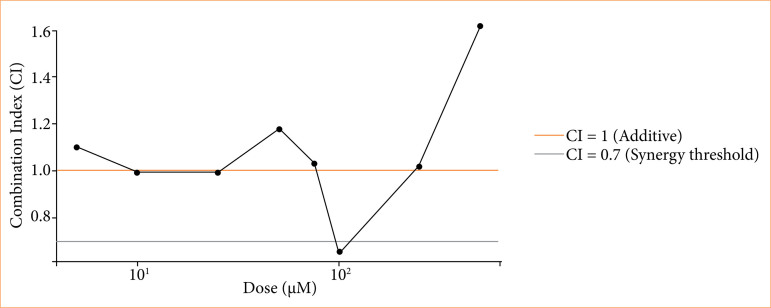
The dose-dependent cytotoxic effects of Res, TMZ, and their combination on U118 GBM cells, as assessed by MTT assay. While both Res and TMZ alone induced a dose-dependent reduction in cell viability, the combination treatment showed a more pronounced decrease, particularly at concentrations above 50 µM. To quantitatively evaluate this interaction, the Chou-Talalay CI was calculated based on inhibition values. The CI values for combined treatment at multiple doses (25, 50, 100, and 250 µM) were all below 0.7, indicating synergy or strong synergy. Res: resveratrol; TMZ: temozolomide; GBM: glioblastoma.

Source: Elaborated by the authors.

The quantitative evaluation of the interaction between Res and TMZ was performed using the Chou-Talalay CI method. As shown in Table 2, CI values were calculated across a range of fixed-dose combinations and revealed a clear dose-dependent synergistic interaction. All tested combinations yielded CI values below 1, indicating synergism. In particular, the CI values at 50 and 100 µM were 0.57 and 0.42, respectively, corresponding to strong synergy, while the CI at 500 µM was 0.31, classified as very strong synergy. These results confirmed that the combination of Res and TMZ produces greater cytotoxicity in U118 GBM cells than either agent alone. This trend is further visualized in Fig. 4, in which CI values decline progressively with increasing concentrations, reinforcing the presence of a true pharmacological synergy rather than an additive response. The findings support the therapeutic potential of Res and TMZ co-administration as a synergistic anti-GBM strategy.

**Table 2:** Combination analysis report: resveratrol + temozolomide.

Dose (µM)	Combo inhibition (%)	Combination index	Combination index interpretation
5	8.9	1.10	Antagonism
10	15.0	1.00	Additive
25	29.8	0.99	Additive
50	49.4	1.18	Antagonism
75	65.2	1.04	Additive
100	80.5	0.66	Synergy
250	89.6	1.02	Additive
500	95.8	1.62	Antagonism

Figure 4 shows the dose-dependent CI values for the combination of Res and TMZ. CI values below 1 indicate synergy, with the most pronounced synergistic effect observed at higher concentrations.

These findings support a synergistic effect of Res and TMZ in suppressing GBM cell proliferation, particularly at higher concentrations.

### Gene expression findings

**Figure 5:**
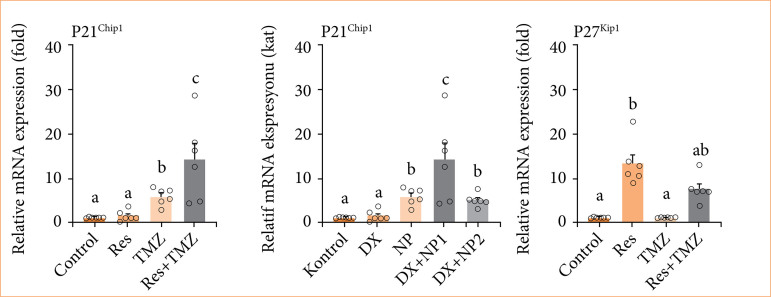
Relative fold increase values of p21, p27 and p53 gene expressions 48 h after single and combined application of Res and TMZ in U118 cell series (data were normalized by β-actin and glyceraldehyde-3-phosphate dehydrogenase (GAPDH) mRNA level by multiple control method, n = 6 data mean ± standard error). Means marked with different letters are statistically different, one-way analysis of variance, Tukey’s honestly significant difference test, p ≤ 0.05. Res: resveratrol; TMZ: temozolomide; Dx: equivalent doses.

Source: Elaborated by the authors.

In the study, Tali image-based cytometry results after Annexin V:PI staining, performed with the Tali apoptosis kit, are presented in Fig. 5. GBM cells were then treated with Res and TMZ for 48 hours, and apoptotic gene expression were analyzed by qRT-PCR. We found that p21 expression was significantly reduced, while p27 and p53 expressions were increased compared to the control group (Fig. 5). Additionally, apoptotic cells were counted by Tali cytometric analysis. Compared to the control group, the number of apoptotic and dead cells significantly increased in the Res and TMZ-treated groups. Cell synthesis was evaluated using the Click-iT Edu imaging kit. Collectively, Res affected cell proliferation and *in-vitro* apoptosis protein levels both upstream and downstream.

### Protein-protein interaction analysis findings

**Figure 6:**
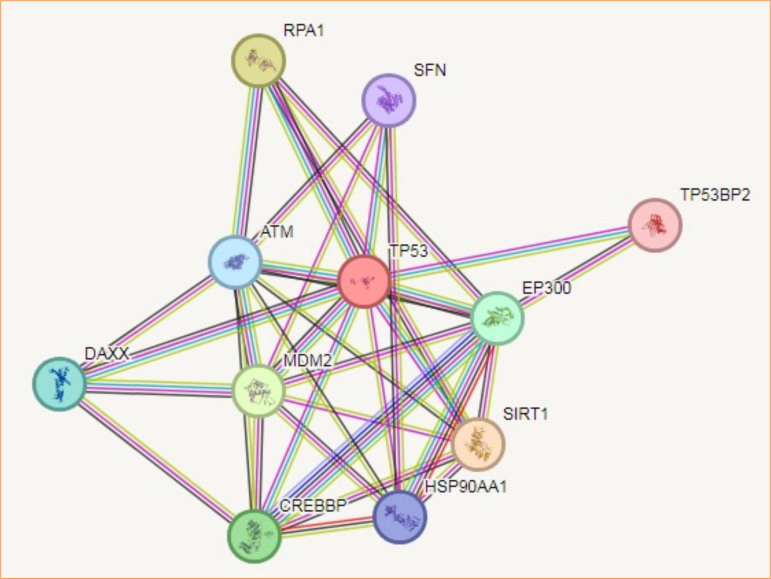
A protein-protein interaction network illustrating interactions between genes relevant to gliobastoma (GBM). TP53, a key tumor suppressor, is centrally positioned, interacting with multiple proteins, including ATM, MDM2, EP300, SIRT1, and TP53BP2. The connections indicate various types of associations, such as co-expression, co-occurrence, and experimental evidence. This dense network suggests that TP53 played a central role in GBM progression through mechanisms involving DNA damage response, apoptosis, and cell cycle regulation. The involvement of additional proteins such as HSP90AA1, CREBBP, and RPA1 underscores complex regulatory functions related to transcriptional control and protein stability in GBM pathophysiology.

Source: Elaborated by the authors.

Predictions from STRING analysis were used to depict protein interactions. The visualization showed 11 nodes and 35 edges (Fig. 6). Based on nodal degree, the following genes were identified as the top 10 central genes: RPA1, SFN, ATM, DAXX, CREBBP, HSP90AA1, SIRT1, EP300, TP53BP2, and MDM2. These targets are hypothesized to be the primary targets in cancers.

### KEGG pathway findings

**Figure 7:**
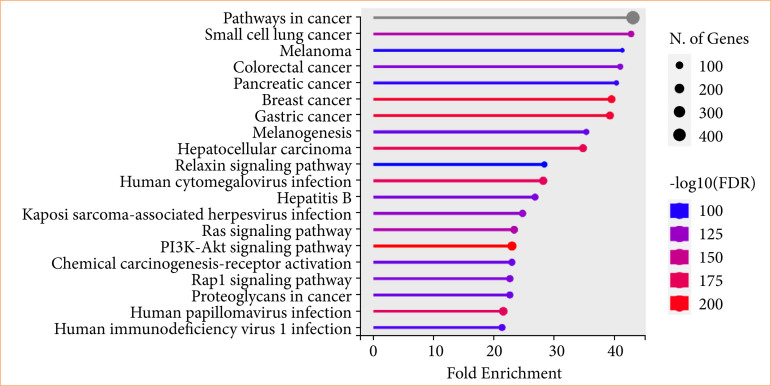
The dot plot represents enrichment analysis for the 400-common compound targets, highlighting significantly enriched pathways related to cancer and infection. The x-axis indicates fold enrichment, while the y-axis lists the pathways. The dot size corresponds to the number of genes associated with each pathway, and the color intensity represents statistical significance [-log10(FDR)], with red indicating higher significance. “Pathways in cancer” shows the highest enrichment, followed by various cancer types, including small cell lung cancer, melanoma, colorectal cancer, pancreatic cancer, and breast cancer. Additionally, key signaling pathways such as PI3K-Akt, Ras, and Rap1, as well as viral infections like human cytomegalovirus, human papillomavirus, and human ımmunodeficiency virus, are significantly enriched. This analysis suggests that the 400-common compound targets played a crucial role in cancer-related mechanisms and infection-associated pathways.

Source: Elaborated by the authors.

KEGG pathway enrichment analysis of target genes was performed with Shiny 0.80 program. The findings showed that 510 genes were involved in the enrichment process and 400 pathways were cancer-related, exhibiting a significant correlation with target genes (*p* < 0.05). Pathways in cancer, small cell lung cancer, colorectal cancer, pancreatic cancer, breast cancer, gastric cancer, melanogenesis, hepatocellular carcinoma, relaxing signaling pathway, the top 10 pathways that occur in cancer are shown (Fig. 7).

### Gene ontology functional enrichment analysis findings

**Figure 8:**
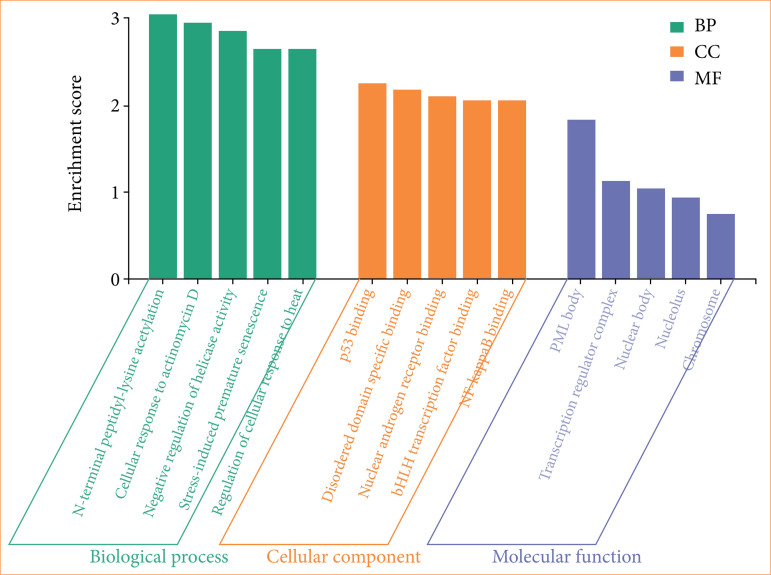
Gene ontology enrichment analysis, categorizing enriched terms into BP, CC, and MF based on enrichment scores. BP terms (green) include “N-terminal peptidyl-lysine acetylation,” “cellular response to actinomycin D,” “negative regulation of telomerase activity,” “stress-induced premature senescence,” and “regulation of cellular response to heat,” indicating processes related to cellular stress and regulation. CC terms (orange) highlight protein interactions and binding sites, including “p53 binding,” “disordered domain-specific binding,” “nuclear androgen receptor binding,” “bHLH transcription factor binding,” and “NF-kappaB binding,” emphasizing transcriptional regulation and signaling pathways. MF terms (blue) include “PML body,” “transcription regulator complex,” “nuclear body,” “nucleolus,” and “chromosome,” pointing to nuclear structures and transcriptional activity. The enrichment scores indicate the relative significance of these functions in the analyzed dataset. BP: biological process; CC: cellular component; MF: molecular function.

Source: Elaborated by the authors.

Analysis findings showed only important functions. Target genes were found to be involved in various cellular components in the BP category, such as N-terminal peptidyl-lysine acetylation, cellular response to actinomycin D, and negative regulation of helicase activity apoptotic process-in terms of cellular components, PML body, transcription regulator complex, nuclear body. It was found that the MF category exhibited roles such as p53 binding, disordered domain specific binding, and nuclear androgen receptor binding (Fig. 8).

## Discussion

The findings of this study provide compelling evidence that Res, in combination with TMZ, effectively induces apoptosis and suppresses proliferation in GBM cells through the activation of key apoptotic signaling pathways. GBM, being one of the most aggressive and treatment-resistant brain tumors, presents significant challenges in clinical management due to its invasive nature and ability to evade apoptosis. Our results demonstrate that Res, a natural polyphenolic compound with well-documented antioxidant and anticancer properties, enhances the therapeutic efficacy of TMZ, the current standard chemotherapeutic agent for GBM. Notably, this synergistic effect is mediated through the modulation of the intrinsic apoptotic pathway, as evidenced by increased expression of pro-apoptotic markers such as Bax and cleaved caspase-3, along with a concurrent decrease in anti-apoptotic proteins such as Bcl-2. Furthermore, the observed inhibition of proliferation was associated with cell cycle arrest at the G2/M phase, suggesting that the Res-TMZ combination disrupts critical regulatory mechanisms involved in tumor cell division and survival. These findings underscore the potential of Res as an adjuvant to TMZ therapy, offering a promising strategy to overcome GBM’s inherent resistance to apoptosis and improve therapeutic outcomes.

Although GBM pathogenesis has been investigated for many years, the molecular mechanisms involved in this process have not been fully explained yet. Many studies have been conducted in the literature to reveal these mechanisms, and these studies are still being intensively carried out. However, to date, a definitively effective treatment protocol for GBM has not been fully established yet. Studies have shown that molecular mechanisms in GBM pathology play a major role during the disease^13^. Natural compounds have gained increasing interest as targets for cancer therapy, because they can target multiple signaling pathways related to tumor progression, metastasis and invasion. Although many natural components related to drug resistance have been discovered, it is emphasized that advanced analyses should be performed in the future to identify them as biomarkers.

Experimental *in-vivo* and *in-vitro* studies have shown that TMZ is effective against a wide range of tumor types. It is known for its clinical efficacy and quality of life improvement in patients with malignant glioma and malignant melanoma. TMZ has been clinically investigated in a wide range of cancers, including advanced soft tissue sarcoma, non-Hodgkin lymphoma, prostate cancer, pancreatic cancer, advanced nasopharyngeal carcinoma, and brain metastases. Malignant gliomas are usually treated with TMZ, but tumor cells are resistant to this chemotherapy. CD133+ cancer stem cells, a population thought to cause tumor chemoresistance, are thought to play a role in this resistance through activation of the Notch and Sonic hedgehog (SHH) signaling pathways. Cyclopamine is a selective blocker of the Hedgehog signaling pathway, which includes key genes that regulate cell proliferation and differentiation^6^,^14^. Cyclopamine, an effective SHH antagonist, binds to the Smo receptor and inhibits the SHH pathway. In fact, a study concluded that cyclopamine treatment disrupts GBM tumor stem cell morphology and reduces tumor stem cell numbers by inhibiting the SHH pathway. SHH is critical in the activation of GBM cancer stem cells, and inhibition of the SHH signaling pathway eliminates GBM tumorigenicity while also increasing the sensitivity of GBM patients to radiotherapy and concomitant and adjuvant TMZ^15^,^16^.

Alkylating agents are the most used chemotherapeutic drugs in the treatment of GBM. Temozolomide is a DNA methylating agent used in the treatment of stage II and stage III glioblastomas. It damages DNA by adding a methyl group to the O6 position of guanine in the most critical region. This damage causes apoptosis by stopping cancer cells in the G2/M cell cycle^17^. TMZ is frequently used in the treatment of recurrent glioma and has an antitumor effect. The appropriate dose given in human GBM is 150 to 200 mg per body surface area daily for five days every 28 days. However, it has been determined that GBM patients show differences in their responses to this TMZ chemotherapy. Although TMZ, which causes DNA damage, is usually the first chemotherapeutic drug used in the treatment of GBM in the clinic, it causes side effects such as myelotoxicity, ulcers, nausea, vomiting, fatigue, and headache. Treatment with TMZ causes O-6-methylguanine-DNA methyltransferase upregulation, which increases cancer resistance. Therefore, while TMZ is used as a chemotherapeutic drug, it also paves the way for tumor recurrence^6^,^14^.

Res, a natural polyphenol, has highly effective mechanisms against many cancers^18^. More importantly, it shows very little toxicity and side effects on healthy cells, in contrast to the intensity with which it destroys cancer cells^19^. Therefore, it is thought that it should be investigated alone or in combination with other chemotherapy drugs in the fight against cancer. Studies have shown that Res can increase the sensitivity of tumor cells to chemotherapy drugs such as gemcitabine, vincristine, Adriamycin, and paclitaxel^20^. It has been reported that Res is also used together with TMZ to treat GBMs^21^. However, it is not clear yet whether this combination is effective for all glioma cells, especially for GBM tumors that are double resistant to both TMZ and Res.

Our study aimed to determine the synergistic effects of Res and TMZ against GBMs and the underlying apoptotic mechanisms. The results showed that U118 cells were sensitive to Res or TMZ. Further analysis of the responses of this cell line to different Res, TMZ and their combinations showed that Res (5-500 μM) and TMZ (5118) effectively inhibited the growth and induced apoptosis of cells, suggesting a synergistic effect between them. If the anticancer effect can be achieved at a lower concentration by combination with TMZ, it may provide practicality for *in-vivo* application of Res. It may also provide a solution to the severe side effects of TMZ, such as gastrointestinal^22^ and bone marrow inhibition^23^.

To improve the quality of life of GBM patients after chemotherapy and prolong their survival, it is necessary to prevent migration in tumor cells, overcome resistance to chemotherapeutic drugs, prevent recurrence, and reduce side effects. This depends only on the development of more effective and, when necessary, combined treatment strategies. There is no study in the literature investigating p53 expression after Res and TMZ treatment used in GBM treatment.

In this study, for the first time, the effectiveness of changes in the expression of apoptotic pathway genes after separate and combined application of Res and TMZ in GBM treatment in GBM cell line (U118) was revealed. As a result of our study, significantly increased p21 and p27 levels in the four-week Res and TMZ combined treatment group compared to the other treatment groups (TMZ only, Res only) revealed the effectiveness of the combined treatment. Res applied together with TMZ increased the drug effectiveness in glioma cells. First, it should be clarified through which pathways Res and TMZ exert their inhibitory effects on U118 cells. In addition, the efficacy of Res and TMZ in GBM should be proven by *in-vivo* studies. In the present study, it was determined that apoptotic gene expressions increased significantly after combined treatment with Res and TMZ in the GBM cell line (U118).

Recent studies have increasingly emphasized the therapeutic potential of Res in GBM treatment by targeting various molecular pathways. Res has been shown to inhibit GBM cell proliferation and chemoresistance by suppressing P-glycoprotein and modulating the AKT/PTEN pathway, highlighting its role in overcoming multidrug resistance^24^. Additionally, co-administration of Res with agents like metformin has been proposed to exert synergistic antitumor effects by targeting cellular metabolism and epigenetic regulation in GBM cells^25^. Inflammation, a hallmark of GBM pathophysiology, can be also attenuated by Res through inhibition of the NLRP3 inflammasome via JAK2/STAT3 signaling^26^. Mechanistically, Res modulates the Akt/GSK-3β/NF-κB axis, contributing to reduced proliferation and enhanced apoptosis in glioma cells^27^. Furthermore, combined application of Res with Notch inhibitors has demonstrated enhanced autophagic and apoptotic cell death, suggesting its use in combinatorial regimens^28^. A recent study reported that Res, when combined with 5-fluorouracil, downregulates TRPM2 and β-catenin, leading to decreased GBM cell viability^29^. Importantly, Res targets both AKT and p53 in GBM stem-like cells, suppressing tumor growth and invasiveness, which underscores its role in targeting the glioma stem cell population^30^. Finally, reviews summarizing preclinical evidence confirm the multifaceted antitumor mechanisms of Res in brain cancer, including apoptosis induction, oxidative stress modulation, and epigenetic reprogramming^31^. These findings collectively reinforce the rationale for integrating Res into GBM treatment protocols, either alone or in combination with standard chemotherapeutic agents.

Despite the promising findings of this study, several limitations should be acknowledged. First, the experiments were conducted *in vitro* using GBM cell lines, which may not fully recapitulate the complexity of the tumor microenvironment *in vivo*. Further validation in animal models or patient-derived xenografts is necessary to confirm the therapeutic potential of Res and TMZ in GBM treatment. Second, the precise molecular mechanisms underlying the apoptotic signaling pathway activation remain to be fully elucidated, requiring additional studies involving genetic or pharmacological inhibition of key regulators. Third, potential off-target effects and cytotoxicity in normal brain cells were not assessed, which is crucial for evaluating the clinical applicability of the combination therapy. Lastly, the study focused on a limited range of concentrations and time points; therefore, a broader investigation of dose-dependent effects and long-term outcomes is warranted. Future research should address these limitations to enhance the translational relevance of our findings.

The findings of this study highlight the potential of Res in combination with TMZ as a promising therapeutic approach for GBM. Future research should focus on validating these results in preclinical animal models to better understand the efficacy and safety of this combination in a more complex tumor microenvironment. Additionally, investigating the molecular mechanisms in greater detail, particularly through transcriptomic and proteomic analyses, could provide deeper insights into the apoptotic signaling pathways involved. Exploring drug delivery strategies, such as nanoparticle-based or blood-brain barrier-permeable formulations, may enhance the bioavailability and therapeutic efficacy of Res in GBM treatment. Furthermore, clinical studies evaluating this combination in GBM patients will be critical for translating these findings into clinical applications. Lastly, assessing potential synergistic effects with other targeted therapies or immunotherapies could open new avenues for more effective GBM treatment strategies.

In this study, we showed that Res prevents the invasion and proliferation of GBM cells and that this effect is achieved by suppressing P53 signaling pathway. With these results, we determined that Res, as a natural phenolic compound, has protective roles in highly metastatic cancer types such as GBM.

## Conclusion

This study demonstrated that Res and TMZ, both individually and in combination, exert potent antiproliferative and pro-apoptotic effects on U118 GBM cells by modulating key apoptotic signaling pathways. Notably, Res significantly increased the expression of pro-apoptotic genes p27 and p53, while downregulating p21, implicating a shift toward cell cycle arrest and apoptosis. Quantitative Chou-Talalay-based CI analysis confirmed a dose-dependent synergistic interaction between Res and TMZ, particularly at higher concentrations, reinforcing their cooperative cytotoxicity. GO and KEGG enrichment analyses further revealed that the co-regulated gene network was highly enriched in cancer-related processes and pathways, including p53 signaling, cell cycle regulation, and DNA damage responses. These mechanistic insights suggest that the combined treatment enhances apoptotic signaling while overcoming TMZ resistance mechanisms commonly observed in GBM. Although promising, these findings require further in-vivo validation, particularly regarding bioavailability, toxicity, and blood-brain barrier permeability. Nevertheless, the Res-TMZ combination represents a compelling therapeutic strategy that warrants advancement toward translational and clinical studies targeting GBM.

## Data Availability

The data will be available upon request.
